# Patent Foramen Ovale Does Not Affect Left Atrial Pressure in Atrial Fibrillation Ablation Patients

**DOI:** 10.3390/jcm15031299

**Published:** 2026-02-06

**Authors:** Marek Kiliszek, Marcin Wańczuk, Beata Uziębło-Życzkowska, Krystian Krzyżanowski, Paweł Krzesiński

**Affiliations:** Department of Cardiology and Internal Diseases, Military Institute of Medicine—National Research Institute, 04-141 Warsaw, Poland; mwanczuk@wim.mil.pl (M.W.); buzieblo-zyczkowska@wim.mil.pl (B.U.-Ż.); kkrzyzanowski@wim.mil.pl (K.K.); pkrzesinski@wim.mil.pl (P.K.)

**Keywords:** patent foramen ovale, atrial fibrillation, heart failure, left atrial pressure, catheter ablation

## Abstract

**Background:** Elevated left atrial pressure is often a consequence of left ventricular diastolic dysfunction. Patent foramen ovale (PFO) with left-to-right shunt could serve as a left atrium unloading factor. The aim of the study was to test whether PFO in patients with atrial fibrillation (AF) is linked to lower left atrial pressure (LAP). **Methods:** A retrospective analysis was performed on consecutive patients undergoing AF ablation from 2019 to 2023. The presence of PFO was assessed with standard transesophageal echocardiography, performed in all patients before ablation. LAP was measured directly in the left atrium just after transseptal puncture. Mean LAP was analyzed. **Results:** A total of 409 patients were included in the analysis, 85 of whom had PFO (20.8%). There were no significant differences between the groups in baseline characteristics such as age, sex, and comorbidities. Overall, 34.4% of patients had a history of heart failure, independent of the presence of a patent foramen ovale (PFO). Mean LAP was not significantly different between patients with and without PFO (*p* = 0.36). **Conclusions:** In patients undergoing AF ablation, more than 20% of patients have PFO. The presence of PFO does not significantly influence LAP, measured directly in the left atrium. The presence of PFO is not linked to a lower prevalence of heart failure.

## 1. Introduction

Heart failure (HF) is characterized by elevated left atrial pressure (LAP), regardless of ejection fraction (EF), compared with healthy individuals [1]. Increased LAP reflects higher left-sided cardiac filling pressures, which are closely associated with symptoms, reduced exercise capacity, and pulmonary congestion, particularly in patients with heart failure with preserved ejection fraction (HFpEF) [2,3].

Catheter ablation of atrial fibrillation (AF) requires access to the left atrium, most commonly achieved via transseptal puncture [4]. Several techniques are available to perform transseptal puncture [5]. The puncture can also be performed using a pressure sensor attached to the needle, allowing for straightforward assessment of when the left atrium has been reached. This approach enables measurement of LAP, which can be used for further analysis [6].

A patent foramen ovale (PFO), a persistent opening in the interatrial septum that allows blood flow between the left and right atria, may serve as a natural interatrial shunt and potentially lower LAP. In a large retrospective study, patients with a PFO had a significantly lower risk of HF hospitalization compared to those without a PFO [7].

These observations suggest that a PFO may attenuate the hemodynamic burden associated with elevated LAP. However, it remains unclear whether the presence of a PFO influences resting LAP in patients with atrial fibrillation (AF). Therefore, we aimed to assess whether patients with a PFO undergoing catheter ablation for AF have lower resting LAP, measured directly before the procedure, compared with those without a PFO.

## 2. Methodology

### 2.1. Study Population

A retrospective analysis was performed, including consecutive patients undergoing AF ablation between 2019 and 2023. Patients were qualified for AF ablation according to current guidelines (recurrent, symptomatic atrial fibrillation) [8]. Patients were included in the analysis if they underwent transesophageal echocardiography before the procedure and subsequently underwent AF catheter ablation. Patients were excluded from the analysis if they had a left atrial appendage thrombus (and could not undergo catheter ablation), an atrial septal defect, or a history of previous procedures (transcatheter or cardiac surgery) involving the intra-atrial septum. No other specific exclusion criteria were applied. Information regarding concomitant diseases was obtained from medical records. The classification of heart failure subgroups (heart failure with reduced, mid-range, and preserved ejection fraction) was based on current ESC guidelines [9]. Data on natriuretic peptides were not routinely collected and were; therefore, unavailable for analysis. The diagnosis of heart failure with preserved ejection fraction (HFpEF) was not revised in the analysis based on LAP. The study protocol was approved by the Bioethics Committee of the Military Institute of Medicine, National Research Institute, which waived the requirement for informed consent due to the retrospective nature of the study (Resolution nr 18/24).

All the patients underwent AF catheter ablation in conscious sedation (in the majority of cases, RF ablation). At the time of LAP measurement, all the patients were conscious, but sporadically after small doses (1–2 mg) of midazolam. The left atrium (LA) was accessed through a double transseptal puncture using an 8.5F transseptal sheath (either a non-steerable Swartz or a steerable Agilis) and a BRK needle (Abbott Medical, Plymouth, MN, USA). LAP was measured directly, shortly after the transseptal puncture, via a transseptal sheath equipped with a transducer for invasive blood pressure measurement (B. Braun Melsungen AG, Melsungen, Germany) and connected to the electrophysiological system (Boston Scientific Corporation, Marlborough, MA, USA). The transducer was filled with physiological saline and inspected for air bubbles. The system was zeroed in the atmospheric pressure when the end of the transducer was placed at the expected level of the left atrium, usually a few (5–10) centimeters above the operating table. Three values were automatically measured by the electrophysiological system in the LA: highest (LAP max), lowest (LAP min), and mean (LAP mean). In the majority of cases, after connecting the transducer to the transseptal sheath, the values of LAP stabilized after a few seconds and were recorded. If an important respiratory variability was noted, the end-expiratory values were recorded. LAP was measured on the spontaneous rhythm of the patient (sinus rhythm or AF). The mean LAP was used for further analysis. As a rule, PFO was not used as an access to the LA.

### 2.2. Echocardiography Parameters

Transthoracic echocardiograms (TTEs) were performed immediately before ablation using a high-quality echocardiograph (Vivid E95, General Electric, Horten, Norway) [10]. All examinations were analyzed offline by a single experienced echocardiographer, who was blinded to clinical status and accredited by the Section of Echocardiography of the Polish Cardiac Society. During the echocardiographic examination, all necessary measurements of the right ventricle (RV), left ventricle (LV), and left atrium (LA) were obtained.

Transesophageal echocardiography (TEE) was also performed prior to ablation. During TEE, both atria, the interatrial septum, and the left atrial appendage (LAA) were thoroughly scanned with a continuous sweep of the probe from 0 to 180 degrees. Only patients without left atrial appendage thrombus proceeded to catheter ablation.

### 2.3. Assessment of Patent Foramen Ovale

Patent foramen ovale (PFO) presence was assessed retrospectively using transesophageal echocardiography (TEE) performed as part of routine pre-procedural evaluation prior to catheter ablation. TEE examinations were primarily aimed at excluding intracardiac thrombi and were not specifically optimized for detailed PFO diagnostics. PFO was identified using color Doppler imaging at rest. Standard mid-esophageal views were used for evaluation of the interatrial septum, including the bicaval view (approximately 90–110°) and the mid-esophageal four-chamber view (0–20°). Color Doppler settings were optimized for the detection of low-velocity interatrial flow, with a low Nyquist limit typically set at approximately 30–60 cm/s and a focused color box over the interatrial septum. PFO was diagnosed when a distinct color Doppler jet traversing the interatrial septum was visualized in at least one standard view, consistent with interatrial shunting. No agitated saline contrast studies or provocative maneuvers (Valsalva or cough) were systematically performed.

Consequently, PFO assessment was limited to spontaneous interatrial shunting detectable at rest. Detailed anatomic and functional characteristics of PFO, including shunt magnitude, defect diameter, tunnel length, or septal mobility, were not available, precluding stratified or exploratory analyses based on PFO morphology or shunt severity.

### 2.4. Statistical Calculations

The data are presented as mean (standard deviation, SD) or median (interquartile range, IQR), depending on the distribution of the variable. The distribution of continuous variables was assessed with the Shapiro–Wilk test. Categorical variables are presented as numbers (percent, %). The difference between groups was calculated with the Student *t* test or Mann–Whitney U test, according to the distribution of the variables. The Chi-square test and Fisher’s exact test were used to test the differences between groups in categorical variables. A linear regression model predicting LAP was constructed to adjust the effect of PFO for other variables, as well as a logistic regression model predicting LAP > 15 mmHg. Clinically relevant variables included in both models were selected based on previous studies: age, sex, BMI, AF type (paroxysmal, nonparoxysmal), LAVI, left ventricular ejection fraction (LVEF), E/e′, rhythm during measurement (AF vs. sinus rhythm), and concomitant diseases (heart failure, hypertension, diabetes, and previous myocardial infarction) [4,10]. Records with missing data were omitted from all analyses. As missing data comprised approximately 1% of all records, it is unlikely that this affected the results.

In all calculations, a two-tailed *p* < 0.05 was considered statistically significant. Given the available sample size and the observed standard deviation of LAP (5.5 mmHg), the study had 80% power at a two-sided α = 0.05 to detect a between-group difference in LAP of approximately 1.9 mmHg. All the analyses were performed with R (version 4.5.2) [11].

## 3. Results

### 3.1. Baseline Characteristics

The study population consisted of 409 patients. Of these, 85 (20.8%) were diagnosed with PFO. The median age of patients was 65 years, and 38.6% were women. Hypertension was diagnosed in 77.5% of patients, and heart failure in 34.4%. Nonparoxysmal AF (persistent and long-term persistent AF) was in about 47% of patients. Table 1 summarizes the baseline characteristics of the total study cohort, as well as the subgroups with and without PFO. There were no significant differences between the groups in clinical and demographic parameters.

### 3.2. Echocardiography and Direct Left Atrial Pressure Measurement

Echocardiographic parameters were similar between patients with and without PFO (Table 2). Elevated LAP (≥15 mmHg) was observed in 43.8% of the study population. The median LAP in the overall cohort was 15 mmHg (IQR 12–18 mmHg), with no statistically significant difference between patients with and without PFO (*p* = 0.36; Table 2). The mean difference in LAP between patients without and with PFO was 0.62 mmHg (95% CI −0.63 to 1.88), corresponding to a small effect size. The confidence interval was narrow, indicating that any true difference, if present, is likely to be clinically negligible.

### 3.3. PFO in Lap Prediction

A linear regression model for predicting LAP was constructed. After adjustment for other variables (as described in the statistical methods), PFO remained non-significant (*p* = 0.09). In a logistic regression analysis of predictors of LAP ≥ 15 mmHg, after adjusting for all variables that could potentially influence LAP (as in the linear regression model), PFO again remained non-significant (*p* = 0.55).

### 3.4. Selected Subgroup Analysis

Several subgroups in which an unloading effect of PFO would be most likely were identified: patients with elevated LAP, non-paroxysmal EF, enlarged LA (LAVI ≥ 34), and those who were obese (BMI ≥ 30). In none of these groups was the effect of PFO significant (see Table 3). An analysis of subgroups of patients with LAP measured during sinus rhythm and atrial fibrillation was also performed.

## 4. Discussion

Our study demonstrates that there is no difference in mean LAP, measured directly in the left atrium, between patients with and without PFO undergoing AF ablation. The proportion of patients with PFO in our study is consistent with previously published data [7,12].

Increased LAP reflects higher left-sided cardiac filling pressures, which are closely associated with symptoms, reduced exercise capacity, and pulmonary congestion. Given these pathophysiological mechanisms, lowering LAP has become a therapeutic target in HF [13]. One of the novel attempts involves the creation of an interatrial shunt, which reduces left-sided pressures, thereby alleviating pulmonary congestion. This has demonstrated efficacy in reducing exertional dyspnea in clinical trials [14,15].

Most studies investigating interatrial shunts have reported no significant differences in resting pulmonary capillary wedge pressure (PCWP), which is commonly used as a surrogate of LAP [14,16]. The only exception is the PRELIEVE study, where resting PCWP was 5 mmHg lower after the intervention compared with the control group, although relatively large 12–14 mm balloons were used in that trial [17]. Nevertheless, a statistically significant decrease in PCWP during exercise or passive leg elevation has been demonstrated in the majority of studies investigating interatrial shunts [14,16].

Compared with interatrial shunts, PFOs are typically smaller in diameter (mean 4.9 mm vs. 7–8 mm) [12,14,16], which may be insufficient to produce a significant reduction in LAP at rest. Because LAP measurements were obtained during catheter ablation procedures, assessment of LAP during exercise or passive leg elevation was not feasible. Whether PFO is associated with lower LAP in such conditions remains unknown. However, it is likely that a PFO may play a role in decreasing exercise-induced elevations in LAP. Studies have shown that interatrial shunts significantly reduce exercise-induced LAP, but not baseline LAP [14]. If a PFO functions as an unloading mechanism, this should result in observable clinical incidences of HFpEF exacerbations.

Park et al. conducted a retrospective study to investigate whether the presence of a PFO influences the development of HF. Among 4804 patients without prior HF who underwent transesophageal echocardiography, 981 (20.4%) were identified with a PFO. Over a median follow-up of 3.5 years, patients with a PFO had a significantly lower risk of HF hospitalization compared to those without (adjusted HR 0.65) [7]. This observation suggests a potential protective effect of PFO, despite the absence of a measurable impact on LAP at rest in our study. We did not observe a difference in the frequency of heart failure diagnosis between patients with and without PFO.

A commonly used method for estimating LAP is the measurement of pulmonary arterial wedge pressure (PAWP) during right heart catheterization. Although the upper limit of normal PAWP is considered to be 12 mmHg, the ESC Heart Failure Association suggests a higher threshold for the invasive diagnosis of heart failure (HF) with preserved ejection fraction (HFpEF) (PAWP ≥ 15 mmHg) [18]. The observed discrepancy between the proportion of patients diagnosed with heart failure (34.4%) and those with elevated LAP (≥15 mmHg, 43.8%) suggests that HFpEF may have been underdiagnosed in our cohort.

Atrial fibrillation and heart failure care are moving into a new era where artificial intelligence is becoming a central driver of progress. Machine learning models are already improving early detection, refining risk prediction, and personalizing ablation strategies in ways that traditional clinical tools cannot match. By collecting a vast array of details—including comorbid conditions, echocardiographic parameters, and invasive measurements—AI is expected to support real-time decision-making in the electrophysiology lab and guide long-term management with unprecedented precision. The future of AF treatment will likely be defined by this fusion of advanced analytics and clinical expertise, accelerating the development of truly individualized therapy [19].

### Strengths and Limitations

A major strength of this study is the relatively large cohort size, comprising 409 patients. In addition, LAP was assessed using direct invasive measurements rather than indirect estimates, increasing the accuracy and reliability of the hemodynamic data.

However, the retrospective design of the study introduces potential selection bias and limits causal interpretation. LAP measurements were obtained exclusively under resting conditions and, therefore, may not reflect dynamic changes during physiological stress. Finally, PFO characteristics, including defect diameter, were not evaluated, limiting assessment of their potential influence on hemodynamic parameters.

## 5. Conclusions

In patients undergoing ablation due to AF, there is no difference in directly measured mean LAP at rest between patients with and without PFO.

## Figures and Tables

**Table 1:** Baseline characteristics of the study group.

Characteristic	Total (*n* = 409)	Without PFO (*n* = 324)	With PFO (*n* = 85)	*p*
Age y (IQR)	65 (58–71)	65 (58–71)	67 (62–73)	0.058
Sex, Female *n* (%)	158 (38.6)	121 (37.3)	37 (43.5)	0.3
BMI, kg/m^2^ (IQR)	29.4 (26.7–32.8)	29.4 (26.4–32.8)	29 (27–33)	0.73
Hypertension *n* (%)	313/404 (77.5)	247/321 (77)	66/83 (79.5)	0.62
Diabetes *n* (%)	86/404 (21.3)	68/321 (21.2)	18/83 (21.7)	0.92
Stroke *n* (%)	22/404 (5.5)	16/321 (5)	6/83 (7.2)	0.42
Coronary disease *n* (%)	117/404 (29)	93/321 (29)	24/83 (28.9)	0.99
Heart failure *n* (%)	139/404 (34.4)	113/321 (35.2)	26/83 (31.3)	0.51
HFrEF *n* (%)	37 (9.2)	32 (10)	5 (6)	0.27
HFmrEF *n* (%)	40 (9.9)	33 (10.3)	7 (8.4)	0.62
HFpEF *n* (%)	62 (15.4)	48 (15)	14 (16.9)	0.67
**Type of AF**				
Non-paroxysmal *n* (%)	191/405 (47.2)	149/321 (46.4)	42/84 (50)	0.83
mEHRA (IQR)	2 (2–3)	2 (2–3)	2 (2–3)	0.97

AF—atrial fibrillation, HFrEF—heart failure with reduced ejection fraction, HFmrEF—heart failure with mildly reduced ejection fraction, HFpEF—heart failure with preserved ejection fraction, IQR—interquartile range, mEHRA—the modified European heart rhythm association (mEHRA) symptom classification, *n*—number, PFO—patent foramen ovale.

**Table 2:** The results of the echocardiography examination and direct LAP measurement.

	Total (*n* = 409)	Without PFO (*n* = 324)	With PFO (*n* = 85)	*p*
LAVI mL/m^2^ (IQR)	43.7 (35.4–52.6)	43.3 (35.2–52.3)	48.0 (36.6–54.1)	0.08
E/e′ avg (IQR)	8.56 (6.78–10.9)	8.65 (6.8–10.9)	8.35 (6.4–10.7)	0.53
EF % (IQR)	58 (50–64)	58 (50–64)	59 (50–63)	0.32
LVDd mm (IQR)	50.9 (48.0–55.0)	51.0 (48.0–55.0)	50.0 (48.0–55.0)	0.70
RVDd mm (IQR)	31.0 (29.0–33.0)	31.0 (29.0–33.0)	32.0 (29.0–34.0)	0.14
Mean LAP mmHg (IQR)	15 (12–18)	15 (12–19)	14 (12–18)	0.36
LAP ≥ 15 mmHg (%)	179 (43.8)	144 (44.4)	35 (41.2)	0.63

EF—ejection fraction, LAP—left atrial pressure, LAVI—left atrial volume index, LVDd—left ventricular end-diastolic diameter, PFO—patent foramen ovale, RVDd—right ventricular end-diastolic diameter.

**Table 3:** Analysis of LAP in selected subgroups of patients.

	Without PFO	With PFO	*p*
LAP ≥ 15 (*n* = 204)	18 (16–22)	18 (16–19.8)	0.20
HFpEF (*n* = 62)	16 (13–20.5)	17 (14.3–18)	0.97
Nonparoxysmal AF (*n* = 191)	16 (13–20.5)	16 (13–18)	0.51
AF during LAP measurement (*n* = 184)	16 (13–20)	16 (13–18)	0.56
SR during LAP measurement (*n* = 220)	13.5 (11–17)	13 (10.8–15.3)	0.46
BMI ≥ 30 (*n* = 183)	15 (13–19)	16 (12.8–18)	0.65
LAVI ≥ 34 (*n* = 320)	16 (12–20)	15 (12–18)	0.50

LAP—left atrial pressure, HFpEF—heart failure with preserved ejection fraction, AF—atrial fibrillation, SR—sinus rhythm, BMI—body mass index, LAVI—left atrial volume index, *n*—number of patients.

## Data Availability

The data is subject to further analysis and will be available after the publication cycle is completed, upon reasonable request.
